# Lowering N_2_O emissions from soils using eucalypt biochar: the importance of redox reactions

**DOI:** 10.1038/srep16773

**Published:** 2015-11-30

**Authors:** P Quin, S Joseph, O Husson, S Donne, D Mitchell, P Munroe, D Phelan, A Cowie, L Van Zwieten

**Affiliations:** 1 University of New England, Armidale, NSW 2351, Australia; 2 Wollongbar Primary Industries Institute, NSW Department of Primary Industries, 1243 Bruxner Highway, Wollongbar, NSW 2477, Australia; 3 School of Materials Science and Engineering, University of New South Wales, NSW 2052, Australia; 4 CIRAD, UPR AIDA, TAB 115/02 Avenue Agropolis 34398 Montpellier Cedex 5, France and AfricaRice Centre, 01 BP 2031 Cotonou, Bénin; 5 Electron Microscopy Center, AIIM, University of Wollongong, Wollongong NSW, 2522, Australia; 6 Discipline of Chemistry, University of Newcastle, Callaghan NSW 2308, Australia; 7 Department of Physics and Institute for Superconducting and Electronic Materials, University of Wollongong, Wollongong NSW, 2522, Australia; 8 Electron Microscope and X-Ray Unit, University of Newcastle, Callaghan NSW 2308, Australia; 9 NSW Department of Primary Industries, University of New England, Armidale, NSW 2351, Australia; 10 Southern Cross Plant Science, Southern Cross University, Military Road, East Lismore NSW 2480, Australia

## Abstract

Agricultural soils are the primary anthropogenic source of atmospheric nitrous oxide (N_2_O), contributing to global warming and depletion of stratospheric ozone. Biochar addition has shown potential to lower soil N_2_O emission, with the mechanisms remaining unclear. We incubated eucalypt biochar (550 °C) – 0, 1 and 5% (w/w) in Ferralsol at 3 water regimes (12, 39 and 54% WFPS) – in a soil column, following gamma irradiation. After N_2_O was injected at the base of the soil column, in the 0% biochar control 100% of expected injected N_2_O was released into headspace, declining to 67% in the 5% amendment. In a 100% biochar column at 6% WFPS, only 16% of the expected N_2_O was observed. X-ray photoelectron spectroscopy identified changes in surface functional groups suggesting interactions between N_2_O and the biochar surfaces. We have shown increases in -O-C = N /pyridine pyrrole/NH_3_, suggesting reactions between N_2_O and the carbon (C) matrix upon exposure to N_2_O. With increasing rates of biochar application, higher pH adjusted redox potentials were observed at the lower water contents. Evidence suggests that biochar has taken part in redox reactions reducing N_2_O to dinitrogen (N_2_), in addition to adsorption of N_2_O.

It is well established that soils are the dominant source of atmospheric nitrous oxide (N_2_O), though a full understanding of the complex biotic and abiotic factors governing N_2_O production and consumption remains to be achieved^1^,^2^,^3^. Amendment of soil with biochar can lead to a reduction of N_2_O emissions, though under some circumstances biochar amendment has resulted in an increase in N_2_O emissions^4^,^5^. A review of literature published from 2007 to 2013 found that biochar reduced soil N_2_O emissions by a mean 54 ± 6% (95% confidence interval)^6^. Herbaceous- and wood-based biochars were most effective in reducing emissions, while the mean effect of those made from manure was negligible. Cayuela, Jeffery^7^ found that the biochars with a molar H:C_org_ (organic C) ratio of <0.3, indicative of a high degree of aromatic condensation, were more effective in lowering N_2_O emissions than those with a molar H:C_org_ ratio of >0.5. A study by Lin, Spokas^8^ found that biochar application reduced N_2_O production in three soils, apparently through the reaction of the biochar with various N forms (nitrate, nitrite, or N_2_O) and possibly catalytic involvement of iron (Fe). They concluded that biochar reduced N_2_O production in these soils through abiotic (chemodenitrification) mechanisms, and hypothesised that Fe-rich biochar can stimulate the abiotic transformation of nitrate/nitrite/N_2_O to N_2_^8^. Others have also proposed that Fe(II), and perhaps manganese (Mn)(II), play a key role as a catalyst in the abiotic reduction of nitrate (NO_3_^−^) in soils^9^. The variability in response to biochar amendment is well recognised, as are the considerable knowledge gaps that exist in understanding the precise mechanisms through which biochar influences soil nitrogen (N) transformations^4^,^5^,^6^,^10^,^11^.

Diffusion of N_2_O through soil is influenced by soil structural characteristics and moisture content. Soil water impedes gas diffusion and the high water solubility of N_2_O may play a significant role in retarding its movement in the soil^3^,^12^. Biochar application can alter soil structure and thus affect soil functions, enhancing porosity and pore connectivity^13^, water retention, air-filled porosity and gas transport^14^. While biochar is found to affect the N-cycling microbial community, with consequential impacts on microbial N_2_O production^15^, it is also suggested that abiotic factors, specifically adsorption or redox reactions on biochar surfaces, may influence N_2_O emissions^16^,^17^. Biochar has considerable aromatic C content which, in spite of its high stability, has redox activity and mainly functions as a reducing agent^16^. Cayuela, Sánchez-Monedero^18^ propose that biochar can act as an “electron shuttle” and Klüpfel, Keiluweit^19^ found biochars to be redox-active, reversibly accepting and donating up to 2 mmol electrons per gram of biochar. Those produced at highest treatment temperatures (HTTs) of 400–700 °C showing greater activity than those of lower HTTs. It has been noted that Fe minerals may be influential in some of these redox reactions^16^,^20^ and Melton, Swanner^21^ observed that discerning whether biotic or abiotic processes control Fe redox chemistry is a major challenge.

Nitrous oxide was injected into columns containing soil/biochar mixes, 100% biochar and sterilised sand (as a system control) to examine the effect of both water content and biochar amendment on diffusion of N_2_O gas and to determine the importance of adsorption and redox reactions.

## Results

### Analysis of N_2_O data

**Figure 1:**
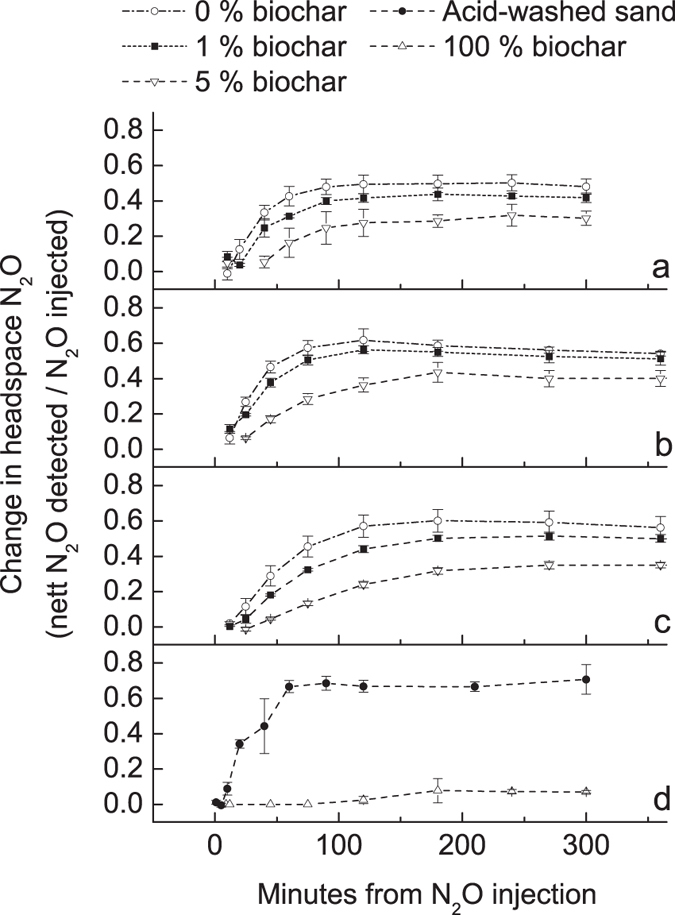
The change in mean headspace N_2_O (injected N_2_O)^−1^ for mean soil water contents of, (**a**) 12% WFPS; (**b**) 39% WFPS; (**c**) 54% WFPS; and also, (**d**) 100% biochar and acid-washed sand (error bars represent ± s.e.m., *n* = 3). At tmax for 12% WFPS the significance of difference in mean nett headspace N_2_O (injected N_2_O)^−1^ between 0 and 1%, 0 and 5% and 1 and 5% biochar was *p* = 0.10, 0.0058 and 0.018 respectively. For 39% WFPS the corresponding values were *p* = 0.31, 0.0054 and 0.016, and for 54% WFPS were *p* = 0.020, 0.00022 and 0.00079. Accounting for N_2_O in WFPS and AFPS, at tmax for 12% WFPS the significance of difference in mean nett (column) total N_2_O content (injected N_2_O)^−1^ between 0 and 1%, 0 and 5% and 1 and 5% biochar was *p* = 0.12, 0.0069 and 0.021 respectively. For 39% WFPS the corresponding values were *p* = 0.34, 0.00076 and 0.018, and for 54% WFPS were *p* = 0.022, 0.00024 and 0.00092.

For each of the 0, 1 and 5% biochar additions to soil, water-filled pore space (WFPS) values of 12 (0.48), 39 (0.47) and 54 (0.50) % were established, hereafter termed low (L), medium (M) and high (H) WFPS (standard error of the mean (s.e.m.) in parentheses, *n* = 3). Moisture contents of the 100% biochar (BC100%) and sand were estimated to be 6 and 3% WFPS respectively. At the end of the sampling periods (tmax) the change in estimated total quantity of N_2_O in air-filled pore space (AFPS) and headspace and dissolved N_2_O in WFPS, divided by the estimated quantity of N_2_O injected (∆N_2_O/inj.N_2_O) for all 0% biochar and acid-washed sand treatments was close to unity (Table 1). Treatments of 1 and 5% biochar had mean values (across all WFPS) of ∆N_2_O/inj.N_2_O at tmax of 0.91 and 0.67 respectively. This suggested that some injected N_2_O was intercepted by these treatments. Treatments were injected with a mean of 22.2 nmol N_2_O (s.e.m. = 1.23 nmol, *n* = 6). When compared with the mean N_2_O intercepted by 0% biochar treatments (−25 pmol), the 1 and 5% biochar treatments significantly lowered N_2_O emitted, by 2.14 and 7.97 nmol respectively (*p* = 0.0094 and *p* = 5.6 × 10^−8^). Although there were differences in ∆N_2_O/inj.N_2_O at tmax between treatments of differing mean WFPS at the same biochar content (Table 1), only that between the 39 and 54% WFPS treatments with 5% biochar was significant (*p* = 0.018). For the BC100% treatments, ∆N_2_O/inj.N_2_O at tmax was only 0.16 (Table 1). The apparent loss of N_2_O within the sampling periods for any treatments containing biochar suggests that some of this gas might have been adsorbed, at least temporarily, or decomposed. Figure 1 shows the mean change in headspace N_2_O (injected mol N_2_O)^−1^ for each treatment. For soil/biochar columns the associated (Fig. 1) caption includes the significance of differences at tmax between treatments of 0, 1 and 5% biochar, based on both headspace N_2_O (injected mol N_2_O)^−1^ and estimated ∆N_2_O/inj.N_2_O. Estimated from headspace N_2_O concentration ([N_2_O]) at tmax, the mean unaccounted N_2_O from headspace and AFPS (injected N_2_O)^−1^ (i.e. N_2_O injected that was ‘missing’ from the combined volume of headspace and estimated AFPS) for 1% and 5% biochar composites was significantly greater than for BC100% treatments per unit weight of biochar (*p* = 0.044 and 0.015 respectively). For treatments of 1% biochar this measure of unaccounted N_2_O was 8.7 (s.e.m. = 1.7, *n* = 3) times greater than the mean for BC100% treatments, and the comparable ratio for treatments of 5% biochar was 4.0 (s.e.m. = 0.37, *n* = 3).

The time to peak headspace [N_2_O] (injected N_2_O)^−1^ was determined for each treatment, with some slight decline expected thereafter due to continued sample removal alone. These values (see Supplementary Table S2 online) reflect the trends seen in Fig. 1, namely that the rate of increase in headspace [N_2_O] (injected N_2_O)^−1^ generally slowed with increase in both biochar content and WFPS. However, the times determined for treatments of medium WFPS, particularly those with 0 and 1% biochar, were somewhat anomalous, being less than the corresponding times for treatments of low WFPS, though not significantly. These differences had parallels in the higher values seen in ∆N_2_O/inj.N_2_O for treatments of medium WFPS than the corresponding treatments of low WFPS, though again the differences being of minor significance for treatments of 0, 1 or 5% biochar (*p* = 0.33, 0.062 and 0.075 respectively). The diffusion coefficient of N_2_O in water is about four orders of magnitude smaller than in air^3^, so increased water content would be expected to retard diffusion. It is surmised that these time-related and headspace [N_2_O] effects, though only minor, could have been an artefact related to the repacking of moist soil, possibly leading to creation of some larger channels in the porespace of those soils than in the soils of low WFPS, so allowing a freer passage of injected N_2_O to the column headspaces. Any such effect would appear to have been overcome by the higher water content of the corresponding soils with high WFPS, where the mean time to reach peak headspace [N_2_O] was greater than for the corresponding drier soils.

There was no increase in headspace [N_2_O] detected in the BC100% treatments until 120 min after N_2_O injection, and little further increase thereafter (Fig. 1). The mean increase in headspace [N_2_O] at tmax in BC100% was only 16.1% of that anticipated from the injected N_2_O (Table 1). Amendment of Ferralsol with 5% biochar has been shown to significantly increase soil porosity, pore connectivity and mean pore radius^13^. Each of these changes would be expected to increase the rate of gas diffusion. Yet, for soils of similar WFPS there was no increase in the rate of headspace N_2_O accumulation with the addition of biochar. On the contrary, as shown in Fig. 1, the rate of and nett change in headspace [N_2_O] (injected N_2_O)^−1^ decreased as % biochar and WFPS increased. These significant differences, and the low headspace [N_2_O] detected in the BC100% treatments, suggest that adsorption and/or decomposition of injected N_2_O is likely to have occurred.

### Modelling of N_2_O data

Modelling of the effects of N_2_O permeation through, and reaction with, a soil sample as a function of WFPS and biochar content can provide insight into the interactions between N_2_O, biochar and soil.

To begin, first assume that there is no interaction at all between the N_2_O and the soil/biochar composite. Under these conditions the processes being observed in the columns can best be regarded as an effusion experiment, where the N_2_O is permeating through the porous structure of the soil/biochar compact as a result of a pressure differential. This process can be modelled based on effusion of gases, which is described by the exponential relationship


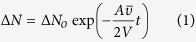


where Δ*N* is the number of gas molecules having moved from one side of the porous medium to the other at time *t*, Δ*N*_o_ is the total number of molecules in the system, *A* is the porous area in the solid composite, 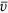 is the average speed of the gas molecules, and *V* is the volume of N_2_O gas before effusion starts^22^. Therefore, the process of effusion is exponential in nature. Equation (1) can be adapted to the following form, using the equivalent terms assessed in this study:


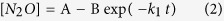


where [N_2_O] is the concentration of N_2_O in the column headspace, A and B are fitting constants, where A–B is the maximum N_2_O concentration change, and k_1_ is the rate constant for effusion. Here k_1_ is related to the terms in the exponential in the previous expression.

Equations (1) and (2) exclude interactions between the N_2_O and the soil/biochar composite through which the N_2_O is effusing. Two possible interactions will now be considered: the dissolution of N_2_O into the water occupying the pores within the soil/biochar composite, and the reaction of N_2_O with the solid components (soil and/or biochar), leading to its decomposition.

The dissolution of N_2_O into the water present in the composite can best be considered as a quasi-equilibrium process where the kinetics of dissolution are much faster than any of the other processes ongoing in the chamber. Given the duration of the experiments, this is a realistic assumption. As such, the total amount of gas phase N_2_O will be lowered by an amount dictated by the solubility of N_2_O in water and the availability of water in the system.

In terms of reaction between the N_2_O and soil/biochar composite little is known about the reaction kinetics, in particular the order of the reaction. Therefore, we have assumed that this is a first order process dependent on the partial pressure of gas phase N_2_O in the system. That is:


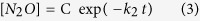


where k_2_ is the rate constant for decomposition, and C is the initial concentration of N_2_O.

Combining all terms together, the resultant expression is:


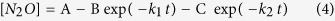


This was then fitted to the experimental data using linear least squares regression (see Supplementary Table S4). A good fit was found between the measured headspace [N_2_O] values and those predicted from the modelling expression, and there was also a fairly close correspondence of times to peak headspace [N_2_O] with those modelled (see Supplementary Table S5). These outcomes offer support to the concepts of diffusion and that adsorption and/or decomposition of injected N_2_O is likely to have occurred.

### pH and Eh

In the soil/biochar composites pH increased with increases in both soil water and biochar content (Table 2). These changes are consistent with decreased soil [H^+^] with increasing water content, and the strong acid-neutralising capacity of the biochar. Values of Eh_pH7_ decreased with increasing WFPS, independent of biochar content, probably due to low oxygen diffusion in water. On the contrary, Eh_pH7_ (Eh corrected to pH = 7) increased with increasing biochar content, most particularly at low WFPS (Table 2). This could lead to the conclusion that biochar addition led to reduction of N_2_O, taking electrons from the composite media and thus increasing its redox potential. However, it is unlikely that the small quantity of N_2_O injected (22 nmol) would be sufficient to promote such significant changes in ~200 g of composite. Initial rapid mineralisation of biochar C and the priming of mineralisation of native soil organic C following addition of biochar have been observed^23^,^24^,^25^. One or both of these processes, which would be increasingly handicapped by increasing WFPS, would seem to be more likely responsible for the changes seen in Eh_pH7_ with increased biochar rate, and would also account for freeing of electrons that might be used in any reduction of N_2_O to N_2_.

**Figure 2:**
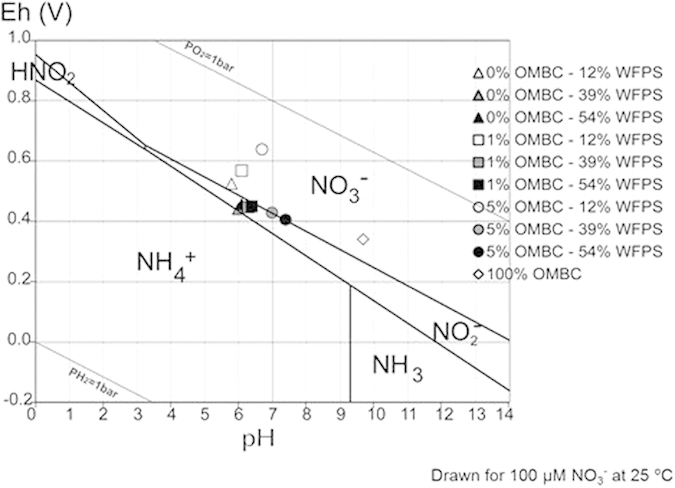
Pourbaix diagram of N representing its various forms in a 100 μM solution at 25 °C as a function of Eh (in V) and pH (diagram drawn using *Medusa software*^60^).

The Pourbaix diagram (Fig. 2) shows that NH_4_^+^ would not be expected to be the dominant form of N in any of the treatments, and that NO_3_^−^ would dominate, especially in the 5% biochar treatment at low WFPS. Yet this was clearly not the case, with NH_4_^+^-N dominant in all soil/biochar treatments. Nevertheless, as anticipated, NH_4_^+^-N increased substantially with increasing WFPS (Table 2). NH_4_^+^-N, relative to pre-packed soil (44 mg kg^−1^), had increased in all medium and high WFPS treatments but decreased at low WFPS. In all soils NO_3_^−^-N had decreased markedly (from 23 mg kg^−1^) to <2 mg kg^−1^ (Table 2). Increased NH_4_^+^-N would not be likely to result from dissimalatory NO_3_^−^ reduction to NH_4_^+^, as this is catalysed by bacteria under anaerobic conditions^26^. Likewise, the abiotic reduction of NO_3_^−^ to NH_4_^+^ involving green rust compounds [Fe^II^_4_Fe^III^_2_(OH)_12_SO_4_ • *y*H_2_O], as proposed by Hansen, Koch^27^, is also only favoured in anoxic environments. The γ-irradiation of soil has been shown to produce an up to 30-fold increase of NH_4_-N_2_^28^ and up to 100% decrease of NO_3_-N_2_^29^. This would appear to be the most likely explanation for the changes observed, and the heightened effect of γ-irradiation on NH_4_-N with higher soil moisture^30^ very strongly supports this conclusion.

### X-ray photoelectron spectroscopy

Results of the analysis of the C and N surface functional groups of the unincubated (original non-irradiated biochar without injected N_2_O – see Method) and aged biochars are presented in Table 3 and Table 4. There was a substantial change in both the concentration of the C functional groups on the surfaces of the biochar pooled from the low, medium and high WFPS 5% treatments (known as LMH5%) and a smaller reduction on BC100%. For LMH5% the concentration of aromatic/aliphatic C = C/C-C/C-H and shake up peaks are much lower, and the BC100% lower, than for the unincubated biochar. The carboxylic and the C-O content had increased for both treatments compared with the unincubated biochar. However an increase in the C = O groups was only measured in the LMH5%. Carbonates were also detected on the surface of the LMH5% from the soil but not on the unincubated biochar or the BC100% treatment. These findings for the biochar from the soil are consistent with those of^16^,^31^ who measured the oxidation of the surface of the biochar after addition to soil. A large increase in the -O-C = N /pyridine pyrrole/NH_3_ was measured in the LMH5%, and a much smaller increase in the BC100%, when compared with the unincubated biochar. A new N group for the LMH5% was detected at 402.56 eV which is often associated with the Pyridine/N-O/Chemisorbed ammonia (NH_3_) and/or the formation of a conjugated N-C-N configuration^32^.

Table 4 reveals considerable differences between the mineral content on the surface of the unincubated biochar and the BC100% and the LMH5%. Iron was present on the surface at 1.3% and silicon (Si) and aluminium (Al) at approximately 7.3% in the LMH5%, whereas no Fe was measured in the unincubated biochar or the BC100%. Silicon and Al were not detected on the surface of the unincubated biochar and only a small amount was detected on the BC100%. Total surface N was increased from approximately 0.9% in the unincubated biochar to 1% in BC100%, and to 1.1% in LMH5%.

### Scanning transmission electron and transmission electron microscopy: examination of the surface and internal structure of aged eucalypt biochar

The structure of the biochar before application to soil is shown in Supplementary Fig. S2. It can be seen that the internal and external C surfaces are typical of a woody biochar that has high C content and a very low mineral content. There were no significant structural or compositional differences noted between the incubated BC100% and the unincubated biochar.

**Figure 3:**
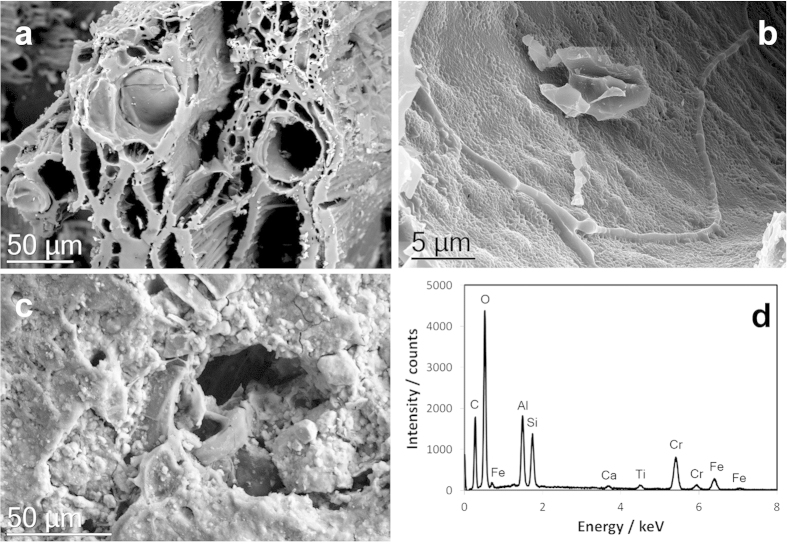
(**a**) SEM image of xylem in the biochar after interaction with soil; (**b**) Internal surfaces of the xylem of the biochar coated in an organomineral film containing significant amounts of Ca and Mg; (**c**) external surface of the biochar and a pore coated with a range of minerals, and (**d**) the EDS spectrum of (**c**).

Figure 3 characterises some of the changes that have occurred on the surfaces of the LMH5%. Some of the internal pores have a layer of organic molecules that are rich in Ca and Mg (Fig. 3b: energy dispersive x-ray (EDS)) and some of the pores have filled with organic matter and mineral matter that is high in Al/Si/O, Fe/O /, Ca/C/O and Ti/O compounds (Fig. 3c). Figure 3d illustrates the range of different mineral phases observed on the surface of the biochar. The EDS data mirrors the survey analysis carried out using x-ray photoelectron spectroscopy (XPS).

**Figure 4:**
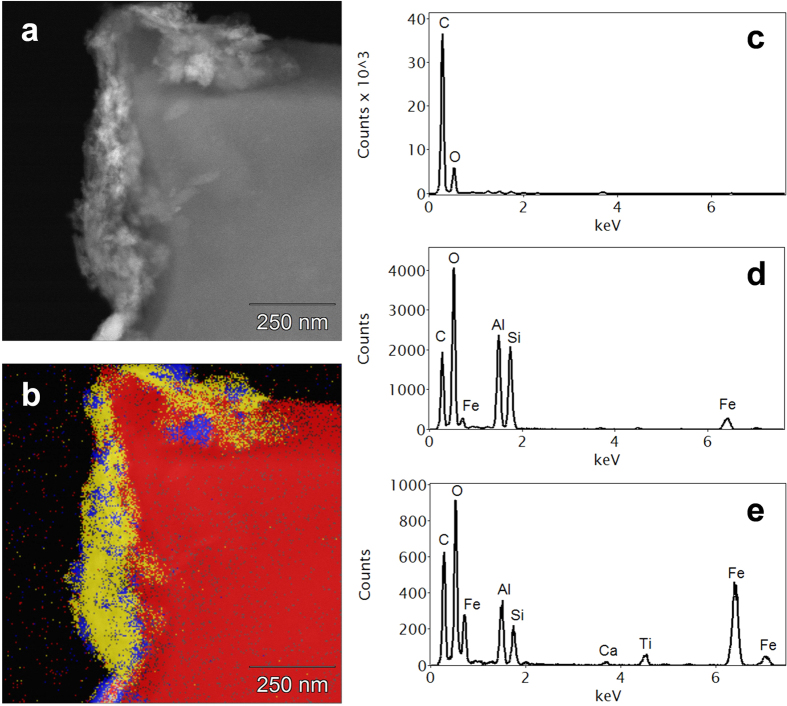
(**a**) HAADF image showing an organomineral layer (bright) coating an external surface of a biochar particle; (**b**) Phase map of (**a**) derived from x-ray microanalysis spectrum imaging, showing three distinct phases; Average EDS spectra of: (**c**) red (biochar) phase in (**b**) containing C and O only; (**d**) yellow (clay) phase in (**b**) containing mainly C, O, Al and Si; (**e**) blue (Fe-rich) phase in (**b**). Both mineral phases [(**c,d**)] have a considerable organic content.

**Figure 5:**
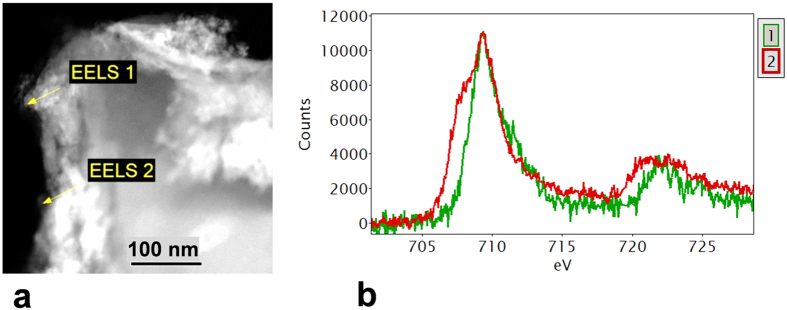
(**a**) STEM HAADF image of biochar with organomineral layer; (**b**) Fe-L_2,3_ EELS spectra (background stripped) were obtained from the points marked in (**a**). The EELS 1 spectrum is characteristic of haematite (Fe III). The spectrum from EELS 2 (red line) shows a pronounced low energy shoulder, suggesting a mixed (II–III) valence state. Note: peak maxima aligned at 709 eV for comparison.

**Figure 6:**
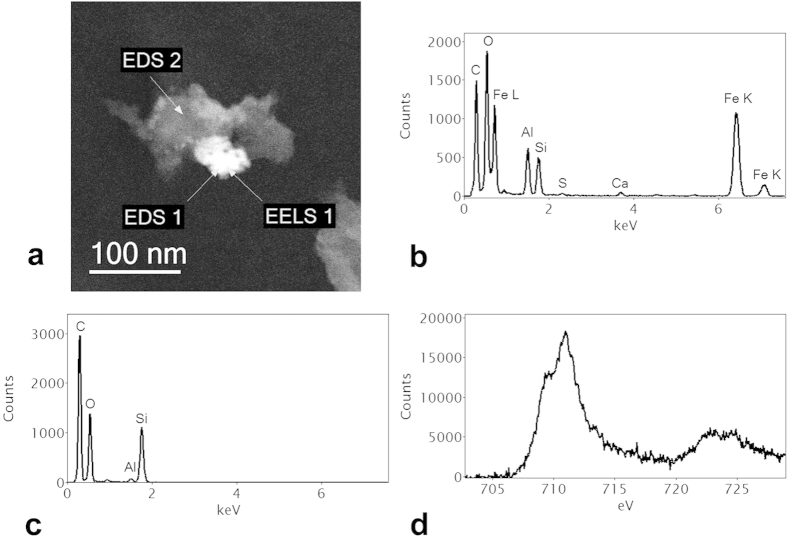
(**a**) STEM HAADF image of edge of biochar showing organomineral phase formed by reaction with soil; (**b**) EDS spectrum of Fe-rich mineral phase (EDS 1); (**c**) EDS spectrum of Si-rich phase (EDS 2); (**d**) Fe-L_2,3_ EELS spectrum (background stripped) at EELS 1 showing a pronounced low energy shoulder on the Fe-L_3_ edge at around 708 eV, characteristic of a mixed (Fe II–III) valence state such as found in magnetite.

Scanning transmission electron microscope (STEM) images and x-ray mapping reveal a nanostructure that is highly heterogeneous. Figure 4 shows a high angle annular dark field (HAADF) STEM image of a section of the biochar particle that has interacted with the soil organic and mineral matter. The associated EDS spectra (Fig. 4) demonstrate the considerable organic content of the mineral phases (see also Supplementary Figure S3). Electron energy loss spectrometry (EELS) of the regions showed strong Fe signals but of varying oxidation state (Fig. 5) with some of the Fe/O phases having an oxidation state of 3 + (haematite) and others a mixed oxidation state of 2+/3+ (possibly magnetite). Transmission electron microscopy (TEM) imaging with selected area electron diffraction indicated that these nanophases could be a mixture of haematite, magnetite and possibly goethite (see Supplementary Figure S4). Figure 6 is an analysis of another interface between a biochar region and a region that has a number of nanophase minerals. On the biochar boundary there are nanophase particles rich in Si/O (probably SiO_2_) and also Fe/O phases that have a mixed (II-III) Fe oxidation state (probably magnetite). In the organomineral phase adjacent to the biochar there are various Al/Si/Ca/Fe/C/S/O nanophase minerals.

## Discussion

In columns containing soil/biochar composites and 100% biochar, nett headspace [N_2_O] was significantly lowered when N_2_O was injected into the profile. This has important consequences for global mitigation options for this greenhouse gas, which also depletes stratospheric ozone^33^. This observation is consistent with studies showing that biochars can lower N_2_O emissions from the soil surface^6^. These previous studies have paid little attention to the different mechanisms involved^10^,^14^. We have shown definitively that that abiotic consumption and/or adsorption of N_2_O is an important mechanism in the studied system.

The interception of N_2_O was not related to soil/biochar moisture content, in the range approx. 12–54% WFPS. Diminishing N_2_O emissions were observed with increasing biochar content in the soil. Cornelissen, Rutherford^17^ examined the sorption properties of a range of softwood biochars. Two, with comparable BET surface areas (176 and 286 m^2^ g^−1^) to the eucalypt biochar, had Langmuir maximum sorption capacities for N_2_O of 47 and 55 cm^3^ g^−1^ respectively at 20 °C under anhydrous conditions, equivalent to 1.95 and 2.29 mmol g^−1^. The BC100% treatments contained a mean 72.7 g of biochar in a close to anhydrous state. It thus seems highly probable that the apparent ‘loss’ of 84% of the mean injected 22.2 nmol of N_2_O from BC100% treatments (Table 1), as calculated from headspace [N_2_O], could be attributed to adsorption. Comparing BC100% with unincubated biochar, there was a small increase from 0.40 to 0.49 atom % of -O-C = N/pyridine pyrrole/NH_3_ revealed by XPS, possibly as a consequence of reaction with (consumption of) N_2_O. The XPS results of the LMH 5% samples indicated that there had been a considerable increase in the -O-C = N /pyridine pyrrole/NH_3_, to 0.77 atom %, and appearance of pyridine/N-O/chemisorbed NH_3_ and/or possibly the formation of a conjugated N-C-N configuration^32^ (Table 3). It is apparent from these increases in N-C/H-N –O-C = N groups that N_2_O had been adsorbed onto the surface of the biochar and could have undergone reactions both with the C and some of the mineral elements (especially Fe nanophase particles identified by TEM) in the biochar. A similar finding re adsorption of N_2_O was reported by Cornelissen, Rutherford^17^. The significantly greater unaccounted N_2_O per unit weight of biochar from headspace and AFPS in soil/biochar treatments than from BC100% also suggests that additional mechanisms may have been responsible for this loss than for that in the BC100%.

The increase in the COOH content (Table 3) and Fe/Al/Si content (Table 4) of the LMH5% is consistent with the findings of Joseph, Camps Arbestain^16^ and Lin, Munroe^34^ who measured the changes to poultry manure/sawdust, greenwaste and paper sludge biochars after 2 years, and poultry manure/sawdust and paper sludge biochars after 3 months respectively in the same Ferralsol used in this study. The latter study found biochars in Ferralsol formed oxidised C surfaces and reacted with soil organic matter. The formation of a porous organomineral layer resulting in the appearance of Fe compounds with Fe^2+^/Fe^3+^ oxidation state indicated that redox reactions^20^ between the N_2_O and the Fe cations may have taken place.

N_2_O is a very strong oxidant. It has a standard reduction potential of 1.77 V, which makes it stronger than O_2_ (1.23 V) and Fe(III) (0.77 V)^35^. Thus it was expected that the 100% biochar would have acted as a catalytic surface to promote the reduction of N_2_O. Our data indicate that biochars will not significantly reduce N_2_O without formation of either redox active organic compounds or organomineral phases high in Fe and other transition metals on their surface. This is consistent with the findings of Carabineiro, Fernandes^36^ who noted that catalysts are required on the surface of activated C to speed up N_2_O reduction reactions at low temperatures.

Reaction of N_2_O might occur with either redox active water soluble organic molecules on the surface of the biochar and/or organic molecules that are deposited from the soil as the organomineral layer is formed during the biochar ageing process. Avdeev, Ruzankin^37^ reported that a range of aromatic and aliphatic compounds are oxidized by N_2_O. They hypothesised that an O atom is transferred through the 1,3-dipolar cyclo-addition of N_2_O to the C = C bond with the resulting intermediate decomposing to yield a ketone and N_2_. Biochars contain a range of aromatic and non-aromatic compounds on their internal and external surfaces. The XPS data presented here, showing substantial increase in ketonic groups in LMH5% (Table 3), indicate that this reaction has taken place.

It is also consistent with the recent research related to the role of Fe^2+^/Fe^3+^ cycling, Eh/pH with N release dynamics and formation and reduction of N_2_O^38^. It should be noted that the soil contains NH_4_^+^ and NO_3_^−^ and these can exist in solution within the water filled pores and from there bind to the surfaces of the biochar. With the injection of N_2_O the following sequence of reactions could take place.


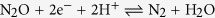



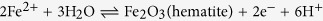



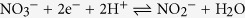



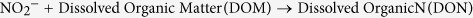


(hypothesised by Li, Yu^39^)


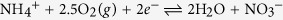


Results of the XPS and the examination of the aged biochar surfaces indicated that there had been significant reactions. Iron has already been reported as a vital key for orchestrating N-transformations^27^,^40^,^41^,^42^. Li, Yu^39^ have shown that reduction and oxidation of N compounds are enhanced when Fe and organic matter are also oxidized or reduced. They refer to this as the “FeIII–FeII redox wheel”. A similar mechanism was found in anoxic environments by Klüpfel, Piepenbrock^43^. Reactions between Fe^2+^ and either NO_3_^−^ or NO_2_^−^ to produce N_2_ (reduced species) are energetically favourable resulting in the formation of iron (oxy-)hydroxide^40^. The addition of amorphous Fe(III)hydroxide (HFO) and, to a lesser extent magnetite, greatly accelerated rates of reaction compared to systems containing Fe^2+^ alone^40^. If N_2_O is adsorbed onto the surfaces of these nanoparticles or if soluble N_2_O surrounds the nanoparticles, catalytic reduction of N_2_O is likely. Sang, Kim^44^ proposed the following reaction mechanism as being the most likely to fit their experimental data for the reduction of N_2_O on Fe exchanged zeolites. This reaction mechanism may also be occurring in the organomineral phases and in the pores of the biochars where there is a concentration of nanophase Fe.


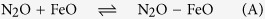



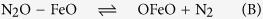



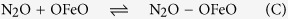



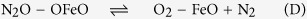



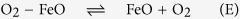


It is true that the system used in this study was not natural, lacking biological activity. Nonetheless, there have been numerous studies investigating the influence of biochar on biological aspects associated with changes to N_2_O emissions from soil, and not all of these changes could be easily explained and indeed, the importance of abiotic reactions has been highlighted in numerous works, e.g. Van Zwieten, Kammann^11^, and Cayuela, Sánchez-Monedero^18^. This work was designed to investigate abiotic reactions following a moderate degree of aging between the biochar and the soil. There is no information on the impact of γ-irradiation on biochar, so this may have impacted the results obtained showing biochar retarded N_2_O movement through the soil profile. However, we deemed it necessary to sterilise the matrix in this way, as other methods (i.e. autoclaving, oven, etc.) could be equally influential in changing properties of biochar.

In summation, eucalypt biochar was shown to lower emissions of injected N_2_O via abiotic mechanisms. In the 100% biochar treatment, the decline in [N_2_0] may be solely the result of adsorption. Given the small quantities of N_2_O injected this could explain why the nett N_2_O in the headspace is substantially less for the 100% biochar than for the soil/biochar treatments, or indeed the sand column system control. The significantly greater decline seen in the composite treatments per unit of biochar, combined with the changes revealed by XPS in biochar from the 5% treatments and changes in Eh_pH7_, suggest very strongly that redox reactions have occurred, reducing a proportion of the intercepted N_2_O to N_2_. There remains much to understand about the importance of abiotic and redox properties in altering soil GHG emissions following biochar addition, yet this offers a significant opportunity to address a globally important issue.

## Materials and Methods

### Soil and Biochar

A Ferralsol^45^ from Wollongbar (28^o^50’S, 153^o^25’E) in north-eastern New South Wales was sieved to ≤2 mm. The soil was rich in Fe sesquioxides (clay content 44.1%; total organic C 4.39%)^46^, with total Fe 8.4%, total Mn 350 mg kg^−1^, total C 4.9%, total N 0.47% and pH of 4.2 in CaCl_2_^47^, NH_4_^+^-N and NO_3_^−^-N contents of 44 and 23 mg kg^−1^ respectively, with NO_2_^−^-N < 0.10 mg kg^−1^ (analytical laboratory accredited to ISO17025). Biochar was obtained from Pacific Pyrolysis, made from the woody residue of *Eucalyptus polybractea* after steam extraction of eucalypt oil. It was produced using a semi-continuous 40 kg h^−1^ pilot unit at a highest treatment temperature (HTT) of 550 ^o^C and heating rate of 5–10 °C min^−1^. Residence time at HTT was 45 min. Measured prior to application the biochar had a pH of 8.65 in CaCl_2_, an acid neutralising capacity of 8.8 (%CaCO_3_ eq.), electrical conductivity of 1.5 dS m^−1^, total C of 70% and total N of 0.81%, NH_4_^+^-N and NO_3_^−^-N contents of 0.49 and <0.2 mg kg^−1^ respectively, Fe content of 0.24% and Mn of 300 mg kg^−1^, and CEC of 17 cmol(^+^)/kg C^48^, a molar H:C_org_ ratio of 0.48 and BET specific surface area of 269 m^2^ g^−1^ 46. The ash content was 11.6%, containing 9,900 and 5,400 mg kg^−1^ of Fe and Mn respectively^49^. (All chemical properties determined by ultimate and proximate analysis using the Australian Standard methods AS 1038.5, AS 1038.6.1 and AS 1038.3).

### Sample packing and irradiation

Air dried soil was sieved to ≤2 mm and biochar to between 250 μm and 2 mm to enhance homogeneity of mixing into small soil volumes. Biochar dosing rates into the columns were 0, 1, 5 and 100% (w/w, dry) biochar, while a system (method check) control of acid-washed sand was also utilized. The columns were 300 mm tall PVC tubes of 37 mm internal diameter, fitted with airtight base and top caps. Each column had a sampling port in the top cap and an injection port in the base cap comprising butyl rubber septa (see Supplementary Figure S1). Soil and soil/biochar mixtures were repacked in 3 equal sections to a depth of 200  mm. Components for each section were individually weighed and distilled water added (by weight) during mixing to homogeneity for the three levels of soil moisture, being 12, 39 and 54% WFPS (*n* = 3) (see Supplementary Table S1 online). Unamended soils were repacked to field bulk density (BD) of 1.02 g cm^−3^. Composites of 1 and 5% biochar were repacked to BDs of 1.00 and 0.93 g cm^−3^ respectively, to account for the lower BD of the biochar (see Supplementary Method online). Columns of 100% biochar and system controls of acid-washed sand were both repacked using gentle tapping (neither with additional moisture) to respective BDs of 0.34 and 1.65 g cm^−3^ (*n* = 3).

The porosities of the soil, 1 and 5% biochar composites and sand were 61.5, 61.9, 63.4 and 37.7% respectively, and that of the biochar from its BD and the density of its solid C fraction^50^ to be between 75 and 80% (see Supplementary Method online). The WFPS of each set of replicate columns was determined as the volumetric water content (see Supplementary Table S1) divided by the relevant porosity.

After repacking, a muslin covered non-absorbent cotton wool plug was inserted into the top of each column to secure the test matrix, and top caps fitted and sealed, enabling the columns to be shipped in an upright position without any disturbance of the packed contents. All columns were weighed and γ-irradiated, using a minimum dose of 25 kGy in order to render the contents abiotic^51^,^52^. The packing was removed from each column inside a UV sterilized biological safety cabinet and the column resealed in the same abiotic environment. Each column was then re-weighed to determine any loss in moisture (none detected). The columns were then incubated at 23 °C for 4 months before N_2_O injection. They were later tested for biological activity (see below). At all stages care was taken to avoid disturbance of the contents.

### Injection of N_2_O and headspace sampling

Injection mixtures (IMs) of N_2_O (>99.8% pure) diluted in N_2_ (99.999% pure) were prepared in 500 mL Tedlar^®^ bags. Samples (*n* = 3) of each IM were injected into pre-evacuated 12 mL Exetainer^®^ vials for later analysis. A 2 mL sample of the IM was injected through the base port using a gas-tight glass syringe with Teflon plunger and a 23G × 1¼” needle inserted to be centre of the column. Immediately prior to injection, and at specific post-injection intervals (see Supplementary Table S3), 2 mL samples of headspace gas were withdrawn through the top port. A tap connected through a side port immediately above the column contents (see Supplementary Figure S1) was opened only during sample withdrawals to maintain atmospheric pressure in the headspace.

Gas samples were analysed according to Van Zwieten, Kimber^47^ (see also Supplementary Method). The rate of diffusion and any abiotic adsorption or degradation of N_2_O gas injected at the base was measured by its accumulation in the column headspace. Pre-injection headspace [N_2_O] was assumed to be in equilibrium with that of air-filled pore space (AFPS [%] = 100 - WFPS), itself assumed to be in equilibrium with N_2_O dissolved in WFPS. From headspace [N_2_O] an estimate was made of the quantity of N_2_O dissolved in WFPS (see Supplementary Method). Total gas pressure within both the headspace of the columns and soil air was assumed to be atmospheric. Dissolved N_2_O was assumed to be in equilibrium with N_2_O in AFPS at the time corresponding to maximum headspace [N_2_O]. For each column the total quantity of N_2_O in headspace, AFPS and WFPS prior to injection was deducted from the same total at diffusive equilibrium, the difference being divided by the quantity of N_2_O injected.

At the end of the experiment, 3 g of substrate was carefully removed from the upper surface of the columns, within the biological safety cabinet, and the columns resealed (see Supplementary Method). These samples were analysed for microbial activity by fluorescein diacetate (FDA) hydrolysis^53^. A range of 0.17–0.34 μg sodium fluorescein (g dry matrix)^−1^ min^−1^ confirmed insignificant microbial activity. The small quantity detected may have resulted from residual enzyme activity^54^.

### pH and Eh

All soil/biochar composites and the BC100% were analysed at the completion of the incubation and gas sampling for NH_4_^+^-N and NO_3_^−^-N content, pH (in H_2_O) by the method of^55^, and redox potential (Eh)^56^ using a hand held ORP meter (Hanna HI 98160) with platinum electrode (Table 2). Redox potentials were transformed^57^,^58^ to correct the Eh to pH = 7 (Eh_pH7_), referenced to the standard hydrogen electrode through the following equation:


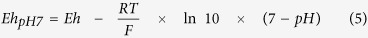


where R is the ideal gas constant (8.31447 J K^−1^ mol^−1^), F the Faraday constant (96485.34 C mol^−1^), and T the temperature (in K).

All Eh measurements were recorded at 25 °C, where


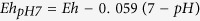
, with Eh expressed in V.

### Examination of the biochar after adsorption of N_2_O

To help determine the possible mechanisms that resulted in the reduction in N_2_O, biochar was studied using a range of electron microscopy and x-ray photoelectron spectroscopy (XPS) techniques. Biochar pieces from different water content columns using the 5% soil/biochar matrix were separated^16^,^34^ and crushed to pass a 0.1mm sieve. Representative samples of the BC100% treatments, and the unincubated biochar (original non-irradiated biochar stored frozen in a sealed container and without injected N_2_O), were also crushed and sieved. Surface functional groups and major mineral elements of the unincubated biochar, the biochar extracted from the soil and the BC100% treatment were measured by XPS analysis (Thermo Scientific ESCALAB250Xi), using a 500 micron diameter beam of monochromatic Al-Kα radiation (photon energy = 1486.6 eV) at a pass energy of 20 eV. The core level binding energies (BEs) were aligned with respect to the C1s BE of 285.0 eV. Examination of over 50 biochar pieces was carried out using a Zeiss Sigma scanning electron microscope (SEM) fitted with a Bruker energy dispersive x-ray analyser as described in Joseph, Graber^59^. To provide detailed microstructural, crystallographic and microchemical analysis both transmission electron microscopy (TEM) and scanning transmission electron microscopy (SEM) was undertaken using JEOL ARM200F aberration corrected TEM fitted with an electron energy loss spectrometer and JEOL EDS detector. To help determine the crystal structure of the mineral phases selected area electron diffraction was carried out in TEM mode (see Supplementary Method for further details of sample preparation and conditions of both TEM and SEM examination).

### Statistical analysis

All statistical comparisons of two groups of data used a two-tailed Welch’s t-test, on account of its suitability for mean values with unequal variance. Unless otherwise stated, any significant difference is based on a 95% confidence level.

## Additional Information

**How to cite this article**: Quin, P. *et al.* Lowering N_2_O emissions from soils using eucalypt biochar: the importance of redox reactions. *Sci. Rep.* **5**, 16773; doi: 10.1038/srep16773 (2015).

## Supplementary Material

Supplementary Information

## Figures and Tables

**Table 1:** The nett increase in total N_2_O content in column air and water relative to N_2_O injected, measured at tmax: 300 minutes for acid-washed sand, 100% biochar and 12% WFPS; 360 minutes for 39 and 54% WFPS (s.e.m. in parentheses, *n* = 3).

% biochar (w/w soil)	Mean WFPS (%)				
	L (12)	M (39)	H (54)	3	6
			∆N_2_O/inj.N_2_O(mol/mol)		
0	0.958 (0.085)	1.020 (0.026)	1.025 (0.115)		
1	0.837 (0.054)	0.970 (0.070)	0.914 (0.040)		
5	0.614 (0.081)	0.772 (0.088)	0.652 (0.029)		
100					0.161 (0.012)
Acid-washed sand				1.056 (0.113)	

**Table 2:** Nitrate- and ammonium-N concentrations, pH and Eh of soil/biochar mixtures after incubation, with Eh values corrected to pH = 7 (s.e.m. in parentheses, *n* = 3).

Biochar (%)	0			1			5			100
WFPS (%)	12	39	54	12	39	54	12	39	54	6
**NH_4_**^** + **^**-N** (mg kg^−1^)	24.7 (0.33) a*c****	72.5 (1.50) a*b*	95.3 (0.67) b*c****j*	23.3 (0.33) d***f****	73.0 (1.15) d***e**k*	94.0 (0.00) e**f****n*	24.0 (0.58) g****h**	77.7 (1.20) g****k*	79.7 (3.18) h**j*n*	<0.3
**NO_3_**^**−**^**-N** (mg kg^−1^)	0.2 (0.03) a**b**	1.2 (0.35)	1.9 (0.56)	0.5 (0.01) a**c**	0.9 (0.24)	1.7 (0.26) c**	0.5 (0.01) b**	1.3 (0.51)	0.9 (0.29)	<0.20
**pH**	5.8 (0) a#b** h#j#	6.0 (0) a#n* q**	6.1 (0.03) b**r* t****	6.1 (0) c*d* h#k#	6.3 (0.03) c*n* p****	6.4 (0.07) d*r*s**	6.7 (0) e**g** j#k#	7.0 (0.03) e**f** p****q**	7.4 (0.03) f**g** s**t****	9.7 (0.1)
**Eh_pH7_** (mV)	454 (3.8) a**f* g*h**	382 (7.0) f*	404 (5.8) a**	515 (4.5) b**c** g*j**	405 (6.9) b**	414 (11.6) c**	621 (12.0) d**e*** h**j**	428 (4.0) d**	429 (14.0) e***	499 (13.3)

Within rows, means accompanied by the same letter are significantly different (*p < 0.05, **p < 0.01, ***p < 0.001, ****p < 0.0001, #*p* = 0).

**Table 3:** C 1s and N 1s bonding state and their relative atomic percentage on the biochar surfaces of eucalypt biochar before addition to columns, extracted from the soil LMH5% treatments and from the 100% biochar treatment, as determined by XPS (regional scan).

Unincubated biochar				Biochar from LMH5% soils		BC100% after addition of N_2_O		
Name	Functional Groups	Peak BE(mV)	At. %	Peak BE(mV)	At. %	Functional Groups	Peak BE(mV)	At. %
C1s A	C = C/C-C/C-H	284.47	68.24	284.38	35.68	C = C/C-C/C-H	284.57	57.10
C1s B	C-O	286.57	6.76			C-O	286.56	12.11
C1s C	C = O	287.97	2.40	286.48	8.04	C = O	287.96	3.35
C1s D	O = C-O	289.17	0.53	287.88	2.89	O = C-O	289.16	3.65
C1s E	Shake up peaks	290.36	6.70	289.08	2.74			
C1s F						Shake up peaks	290.87	4.23
	Carbonate			291.21	1.21			
N1s A	-O-C = N/pyridine pyrrole/NH_3_	400.42	0.40	400.47	0.77		400.93	0.49
N1s B	NH_4_/NH_2_ groups	398.55	0.28	398.69	0.13		399.23	0.25
N1s C	Pyridine/N-O/Chemisorbed NH_3_			402.56	0.11			

**Table 4:** XPS survey of the C, N, O and mineral elements in the three biochar samples (nd = not detected).

Unincubated BC			LMH5%		BC100%	
Name	Peak BE	At. %	Peak BE	At. %	Peak BE	At. %
C1s	284.56	82.38	284.63	42.77	285.39	73.72
O1s	532.42	14.39	532.67	39.40	533.16	19.20
Ca2p	348.08	1.84	347.70	0.75	348.66	2.39
Al2p	nd	nd	75.39	7.37	75.63	0.28
Si2p	103.44	0.51	103.78	7.31	103.89	0.97
Fe2p			712.20	1.32		
N1s	400.14	0.88	400.59	1.07	401.60	0.98
S2p					169.70	0.28
P2p					135.02	0.29
K2s					378.63	0.46
