# Supplementary material for: Lowering N2O emissions from soils using eucalypt biochar: the importance of redox reactions
Source: Sci Rep. 2015 Nov 30;5:16773. doi: 10.1038/srep16773 (PMC4663753; doi:10.1038/srep16773)
Supplement: Supplementary Information [file srep16773-s1.pdf]

Lowering N_2O emissions from soils using eucalypt biochar: the importance of redox reactions.

Authors: P Quin[1, 2*], S Joseph[3, 6, 7*], O Husson[4], S Donne[6], D Mitchell[5], P Munroe[3], D Phelan[8], A Cowie[9], L Van Zwieten[1, 2, 10, #]

[1] University of New England, Armidale, NSW 2351, Australia

[2] Wollongbar Primary Industries Institute, NSW Department of Primary Industries, 1243 Bruxner Highway, Wollongbar, NSW 2477, Australia

[3] School of Materials Science and Engineering, University of New South Wales, NSW 2052, Australia

[4] CIRAD, UPR AIDA, TAB 115/02 Avenue Agropolis 34398 Montpellier Cedex 5, France and AfricaRice Centre, 01 BP 2031 Cotonou, Bénin

[5] Electron Microscopy Center, AIIM, University of Wollongong, Wollongong NSW, 2522, Australia

[6] Discipline of Chemistry, University of Newcastle, Callaghan NSW 2308, Australia

[7] Department of Physics and Institute for Superconducting and Electronic Materials, University of Wollongong, Wollongong NSW, 2522, Australia

[8] Electron Microscope and X-Ray Unit, University of Newcastle, Callaghan NSW 2308, Australia

[9] NSW Department of Primary Industries, University of New England, Armidale, NSW 2351, Australia

[10] Southern Cross Plant Science, Southern Cross University, Military Road, East Lismore NSW 2480, Australia

*P Quin and S Joseph had equal intellectual and experimental inputs into this paper

[#]Corresponding author

Supplementary Information

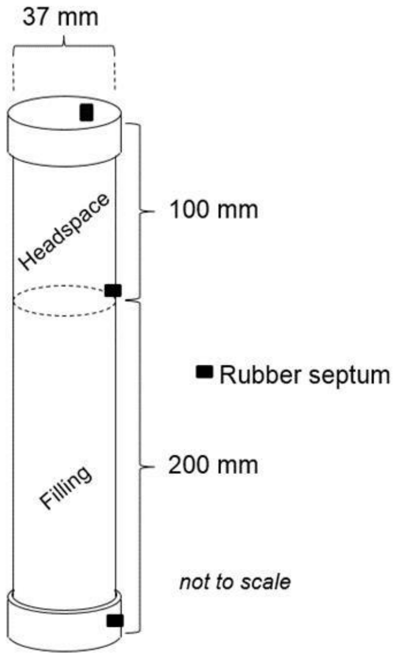

Supplementary Figure S1 | Internal dimensions of columns and locations of rubber septa.

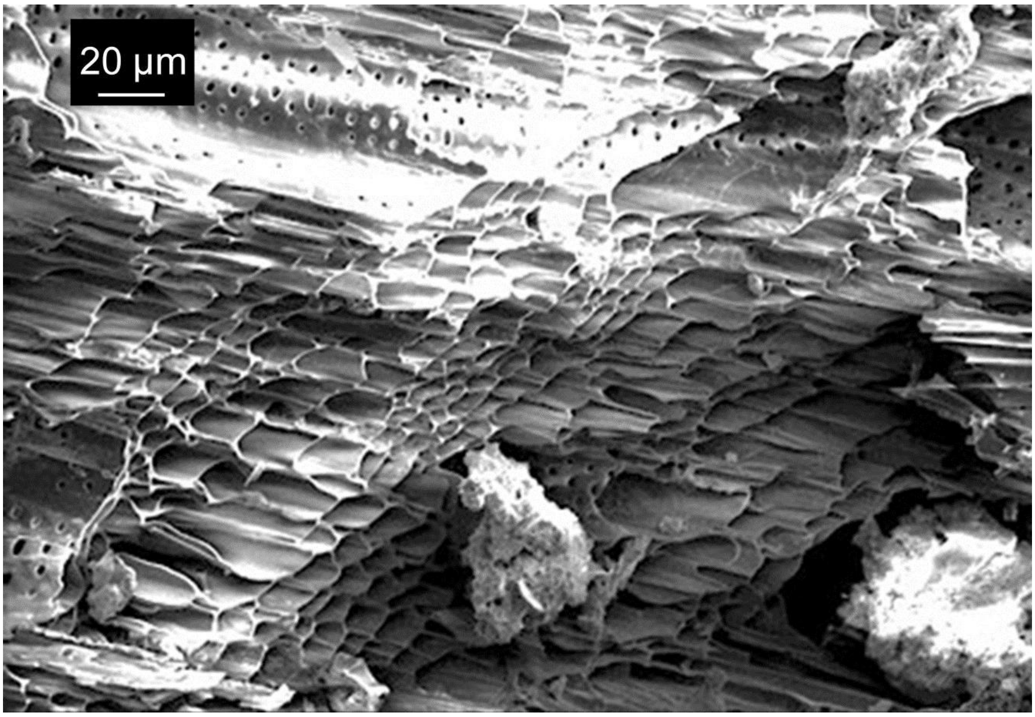

Supplementary Figure S2 | SEM secondary electron image of unincubated eucalypt biochar showing xylem.

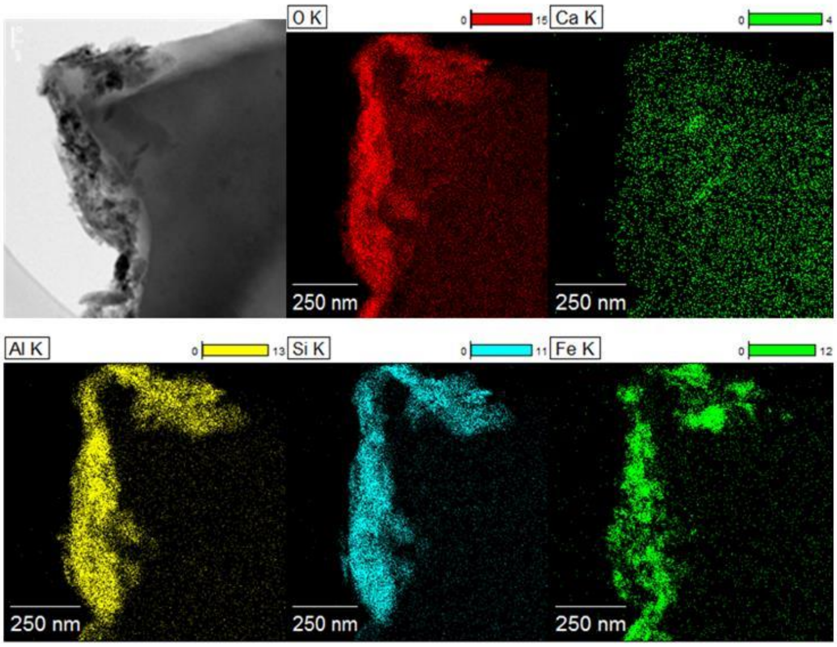

Supplementary Figure S3 | STEM bright field image of an organomineral coated surface of the biochar with associated EDS x-ray maps. The maps differentiate the Fe-rich and Al-Si-rich mineral phases coating the biochar particle.

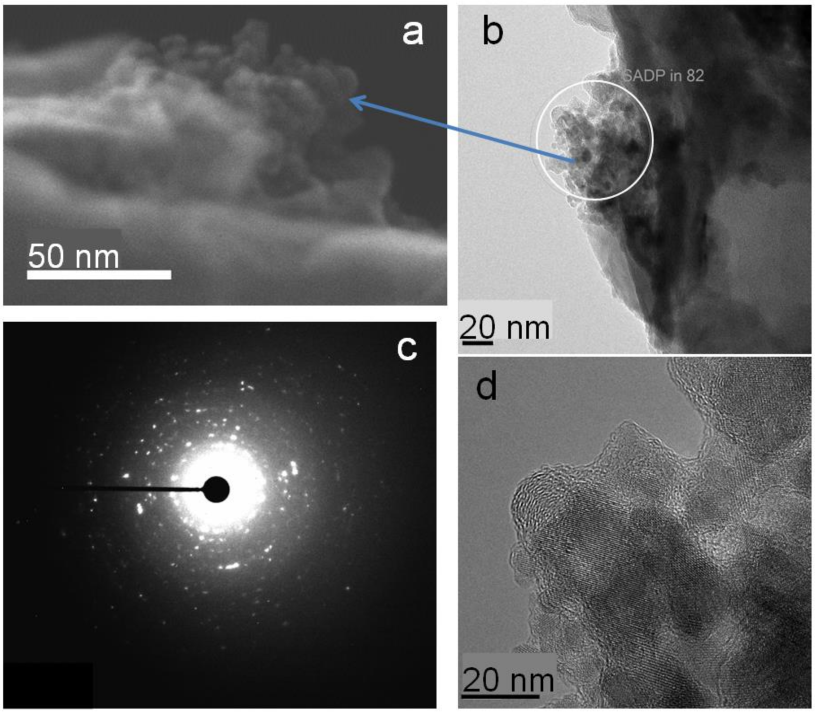

Supplementary Figure S4 | a) Secondary electron image of an organomineral phase on the surface of the biochar (EELS 2 region of Figure 5); b) bright field TEM image of the same area; c) a selected area diffraction pattern of the organomineral phase, and d) high resolution TEM image of the nanophase mineral particles in the edge of the organomineral phase.

Supplementary Table S1 | The estimated volumetric water contents were calculated from measured water content of air-dried components and added distilled water.

% biochar (w/w soil)	Volumetric water content (%)			
	L(air dry)	M	H	
0	7.69	24.01	33.22	
1	7.53	24.89	34.38	
5	7.00	24.65	34.34	
100	2.35	–	–	
Acid-washed sand				2.36

Supplementary Table S2 | The time taken to reach peak headspace concentration of N_2O (s.e.m. in parentheses, *n* = 3).

	Estimated mean WFPS (%)				
	L (12)	M (39)	H (54)	3	6
% biochar (w/w soil)	Mean time to peak headspace concentration of N_2O (minutes)				
0	160 (40)	120 (0)	210 (30)		
1	200 (20)	120 (0)	270 (0)		
5	260 (20)	210 (30)	330 (30)		
100				270 (52)	
Acid-washed sand					120 (46)

Supplementary Table S3 | The post-injection of N_2O sampling times of headspace gas, the range of which was extended when higher WFPS was anticipated to result in slower gas diffusion.

Column contents	Post-injection sampling times (min)
Acid-washed sand	1, 5, 10, 20, 40, 60, 90, 120, 210, 300
100% biochar	1, 2, 4, 6, 9, 12, 18, 26, 35, 50, 75, 120, 180, 240, 300
Group L – mean 12% WFPS	1, 5, 10, 20, 40, 60, 90, 120, 180, 240, 300
Group M – mean 39% WFPS	1, 12, 25, 45, 75, 120, 180, 270, 360,
Group H – mean 54% WFPS	1, 12, 25, 45, 75, 120, 180, 270, 360,

Supplementary Table S4 | The fitted constants A, B and C and rate constants k_1 and k_2 for modelling from Equation 4.

biochar (%)	WFPS (%)	A	B	k_1	− C	k_2
0	12	0.210138	0.767823	0.0342590	0.319180	0.000495145
1	12	0.356426	3.35886	0.0110102	2.95350	0.00933817
5	12	-1.43682	3.20844	0.00327144	4.63635	0.00152126
0	39	0.355073	0.921542	0.0351367	0.315976	0.00144393
1	39	0.423472	0.792305	0.0211095	0.299316	0.00356106
5	39	0.171317	0.614213	0.0177281	0.285448	0.000438543
0	54	0.360409	0.878152	0.0173103	0.361459	0.00140208
1	54	0.277527	0.778682	0.0126906	0.373807	0.00127144
5	54	0.226885	0.587537	0.00972940	0.215826	0.00102071

Supplementary Table S5 | Comparisons of parameters measured and predicted by model (s.e.m. in parentheses, *n* = 3).

biochar (%)	WFPS (%)	Mean peak headspace [N_2O] (nett mol/mol injected)		Time to peak headspace [N_2O] (min)	
		Measured	Predicted by model	Measured	Predicted by model
0	12	0.50 (0.05)	0.50	160 (40)	151
1	12	0.44 (0.04)	0.44	200 (20)	175
5	12	0.32 (0.06)	0.32	260 (20)	227
0	39	0.62 (0.06)	0.61	120 (0)	127
1	39	0.56 (0.02)	0.57	120 (0)	157
5	39	0.44 (0.06)	0.42	210 (30)	258
0	54	0.60 (0.06)	0.61	210 (30)	214
1	54	0.51 (0.02)	0.52	270 (0)	266
5	54	0.35 (0.02)	0.36	330 (30)	374

Supplementary Method

Column preparation and gas sampling

Each column was fitted with sampling ports, 205 mm above the base and in the upper surface of the top cap, and an injection port 5 mm above the base, all fitted with butyl rubber septa (Figure S1). All water was assumed to have a density of 1.00 g cm^{-3}. The bulk density (BD) of sieved biochar, measured by the method of Balco and Stone [1], was 0.338 g cm^{-3}.

The porosity of the soil, assuming a mean particle density of 2.65 g cm^{-3}, was estimated as $(1 – BD/2.65) \times 100 = (1 – 1.02/2.65) \times 100 = 61.5$ %. The porosity of the biochar was estimated from its BD and the density of its solid C fraction [2] to be between 75 and 80 %, and thus taken to be 77.5 %. Porosities of composites were calculated from estimated porosities of the components.

After repacking, the top surface of the contents of each column was covered with a 37 mm diameter PVC disk, which had string attached to facilitate later removal. On top of these was packed non-absorbent cotton wool inside muslin bags, tied at the neck with string. Finally, the top caps were fitted, sealed to each column with silicone tape and each column weighed. On return from γ-irradiation and removal of temporary packing each column had an internal headspace of 129.3 mL.

Two 2 mL samples of each injection mixture (IM) were taken prior to sample injection into columns. A third 2 mL sample of the IM was also taken when time permitted, generally about 40 min after the first injection. Prior to analysis all samples had an additional 23 mL of N_2 gas added, increasing the pressure in each vial to >2 atmospheres in order to suit the analytical equipment. Immediately prior to injection of any IM a 2 mL sample of headspace gas was withdrawn from each column. The air used to flush the acid-washed sand column

between uses was passed into the column via the bottom septum and allowed to exit via the top septum.

From headspace [N_2O] an estimate was made of the quantity of N_2O dissolved in water-filled pore space (WFPS), with solubility estimated as $C_{aq} = K_0 \times P_{N_2O}$, where C_{aq} is the concentration of the gas in water (mol L^{-1}), P_{N_2O} the measured partial pressure of the gas (atm.) in soil air samples and K_0 the equilibrium constant at the mean laboratory temperature of 24.33 ^oC (range 22.9–26.9 ^oC, standard error of the mean = 0.014 ^oC, n = 2370), having a value of 2.55×10^{-2} mol L^{-1} atm^{-1} [3]. Total gas pressure within both the headspace of the columns and soil air was assumed to be atmospheric and the molar volume of N_2O at this pressure to be 24.2 L [3].

The samples analysed for microbial activity by fluorescein diacetate (FDA) hydrolysis were taken from columns representing biochar content and WFPS of 0 % and 12.5 %, 0 % and 54.0 %, 1 % and 40.0 %, 5 % and 10.9 % and 5 % and 38.4 % respectively.

TEM sample preparation and examination

Small fragments of biochar were placed in an agate mortar and lightly ground in ethanol to produce a suspension. A small volume of the suspension was withdrawn with a pipette and a single drop placed on a holey carbon support film supported on a standard copper TEM grid. The specimen was allowed to air dry. Examination was carried out using a JEOL ARM200F probe-corrected scanning transmission electron microscope (STEM) operating at 200kV. Imaging was carried out using both high angle annular dark field (HAADF) and bright field modes. The former providing atomic number contrast and the latter diffraction contrast. Images were acquired using a Gatan DigiScan scan controller system managed by Gatan's

DigitalMicrograph software. The microscope was fitted with a cold field emission electron source and a Gatan Quantum imaging filter for electron energy loss spectroscopy (EELS). Energy resolution in EELS was 0.7eV with the probe conditions used. The microscope was also fitted with a JEOL large area (1sr) energy dispersive spectrometer (EDS) for x-ray microanalysis. Point analyses were carried out using the Gatan EDS plugin within DigitalMicrograph, while spectrum imaging/x-ray mapping was carried out using the Noran System Seven EDS software platform.

SEM analysis

The sample was mounted on SEM stub with carbon tape and was chromium coated. This coating was used as there is little interaction with any of the major elements that are observed on the surfaces of biochar. The EDS spectrum was acquired at 15kV and a working distance of 8 mm. The acquisition time depended on the signal intensity but was normally 120 seconds.

References

1. Balco G, Stone JO. Measuring the density of rock, sand, till, etc. University of Washington, Cosmogenic Isotope Laboratory/chemistry (2003) Available at: http://depts.washington.edu/cosmolab/chem/density_method.pdf (Accessed: 4th August 2011). Adaption of glass bead method.

2. Downie A, Crosky A, Munroe P. Physical Properties of Biochar. In: Biochar for Environmental Management: Science and Technology (eds Lehmann J, Joseph S). Earthscan (2009).

3. Weiss RF, Price BA. Nitrous-oxide solubility in water and seawater. *Mar. Chem.* **8**, 347-359 (1980).
